# Changes in hospital costs after introducing an intermediate care unit: a comparative observational study

**DOI:** 10.1186/cc6903

**Published:** 2008-05-15

**Authors:** Barbara CJ Solberg, Carmen D Dirksen, Fred HM Nieman, Godefridus van Merode, Martijn Poeze, Graham Ramsay

**Affiliations:** 1 Staff Department of Research, Care and Education, Maastricht University Hospital, P. Debyelaan 25 6229 HX Maastricht, The Netherlands; 2 Clinical Epidemiology and Medical Technology Assessment (KEMTA), P. Debyelaan 25 6229 HX Maastricht University Hospital, Maastricht, The Netherlands; 3 Department of Health Organisation, Policy and Economics (BEOZ), University of Maastricht, P.O. Box 616, 6200 MD Maastricht, The Netherlands; 4 Department of Surgery, P. Debyelaan 25 6229 HX Maastricht University Hospital, Maastricht, The Netherlands; 5 Current address: West Hertfordshire NHS Trust Hillfield Road, Hemel Hempstead, HP2 4AD, UK

## Abstract

**Introduction:**

The high cost of critical care resources has resulted in strategies to reduce the costs of ruling out low-risk patients by developing intermediate care units (IMCs). The aim of this study was to compare changes in total hospital costs for intensive care patients before and after the introduction of an IMC at the University Hospital Maastricht.

**Methods:**

The design was a comparative longitudinal study. The setting was a university hospital with a mixed intensive care unit (ICU), an IMC, and general wards. Changes in total hospital costs were measured for patients who were admitted to the ICU before and after the introduction of the IMC. The comparison of interest was the opening of a six-bed mixed IMC.

**Results:**

The mean total hospital cost per patient increased significantly. Before the introduction of the IMC, the total hospital cost per patient was €12,961 (± €14,530) and afterwards it rose to €16,513 (± €17,718). Multiple regression analysis was used to determine to what extent patient characteristics explained these higher hospital costs using mortality, type of stay, diagnostic categories, length of ICU and ward stay, and the Therapeutic Intervention Scoring System (TISS) as predictors. More surgical patients, greater requirements of therapeutic interventions on the ICU admission day, and longer ICU stay in patients did explain the increase in hospital costs, rather than the introduction of the IMC.

**Conclusion:**

After the introduction of the IMC, the higher mean total hospital costs for patients with a high TISS score and longer ICU stay explained the cost increase.

## Introduction

The high costs of critical care have resulted in strategies for improving intensive care utilisation and a more effective triage [1-3]. Admitting low-risk or chronically critically ill patients to intermediate care units (IMCs) rather than an intensive care unit (ICU) has been proposed as an effective and efficient strategy [4,5]. Reports on the cost-effectiveness of introducing an IMC show variable results [6-10]. Several retrospective studies indicate reduced total costs of specialised care, which are achieved by reducing nursing procedures and laboratory tests. However, another trial failed to show a significant effect on costs. Some reports show increased ICU costs during increased bed availability. In addition, whether introducing IMCs reduces total hospital costs is unknown. To study the effects on ICU utilisation and costs, an IMC was opened at our institution. The aim of the present study was to investigate whether introducing an IMC would result in lower total hospital and special care costs. We expected that the improved effective care would decrease these costs.

## Materials and methods

### Design

The study was designed as a comparative longitudinal study that compared hospital costs as well as clinical and hospital data of patients who were admitted to the ICU before (pre-IMC period) and after (IMC period) the introduction of the IMC. The total study period was 20 months: the pre-IMC period was 12 months and the IMC period was 8 months. The institutional review board approved the study. The requirement of informed consent was waived because the IMC was included in the usual care and no extra variables had to be collected.

### Patient population

The study population consisted of two groups of patients who were admitted to the ICU before and after the opening of the IMC. A total of 329 patients were randomly selected by computer from the group of 795 patients during the pre-IMC period. All patients admitted to the ICU in the IMC period were consecutively enrolled (n = 457). Patients admitted to the ICU who came from the IMC ('step-up' patients) were excluded from the analysis (n = 12) to avoid other specific patient characteristics from influencing the total hospital costs. The IMC was only a step-down facility at the beginning, and only at two months within the IMC period were step-up patients also admitted.

### Setting

The IMC was opened at University Hospital Maastricht, adjacent to the medical-surgical ICU. The IMC had six beds in an open concept without isolation facilities. The general ICU was divided in two units, one of eight beds and one of nine beds. After the IMC had been opened, one ICU bed was closed (reducing the total to 16). The ICU and the IMC were supervised and staffed by the same team of critical care physicians, who were available in the ICU and IMC 24 hours/day, 7 days/week. To optimise the efficiency of the IMC, bed management was placed under the supervision of the medical ICU/IMC team. The nursing staff for the IMC was a newly engaged team and was supplemented with one ICU nurse per shift. The nursing team were given a special training course. The ratio of nurses to patients in the IMC was 1:2 on the day shift and 1:3 for evenings and nights versus 1:1 in the general ICU and 1:8 in the wards.

Admission and discharge criteria for the ICU and IMC were based upon the criteria defined by Knaus and colleagues and Keenan and colleagues [11-14]. Identification of ICU patients was based upon interventions that could not be performed outside the critical care unit. These interventions were classified as 'active treatment' according to Therapeutic Intervention Scoring System (TISS-28) variables such as mechanical ventilation and left atrium monitoring. Non-active treatment variables represented interventions that could be carried out in the IMC. Intermediate care was defined as a level of care between intensive care and care on the general ward.

Cardiac patients were admitted to other specialised ICUs (cardiac ICUs). These units and the recovery (24 hours) served as overflows for general ICU patients when the general ICU was fully occupied. ICU patients admitted to recovery room and cardiac ICUs were excluded from the analysis. Neurosurgical and cardiac-surgical patients were transferred from the ICU to a specialised IMC integrated into the general ward.

### Data collection

The data collected for each ICU patient included the following: age, gender, length of stay (LOS) (ICU, IMC, and ward), mortality (ICU and general ward), type of stay (non-surgical versus surgical), TISS-28 score, severity of illness, and diagnostic category. The TISS-28 has been widely applied to assess the nursing workload and interventions in ICUs and IMCs and was used in our study to take measurements on a daily basis via an electronic patient-data management system [15]. The TISS-28 is also used to categorise the level of care that patients require [16]. The TISS-28 scores were assessed daily. The severity of illness was measured by the Acute Physiology and Chronic Health Evaluation II (APACHE II) scoring system on the admission day. The severity of illness was measured by the APACHE II. The diagnostic category was defined according to the diagnostic classification in APACHE II (neurological, neurosurgical, respiratory, gastrointestinal, cardiovascular, multitrauma, sepsis, renal, metabolic, and haematological) [17].

### Cost analysis

The costs were analysed from the hospital point of view. The hospital costs were calculated from admission to the ICU to discharge from the hospital. The hospital costs for patients from the pre-IMC period were compared with hospital costs of patients from the IMC period. Costs were divided into costs of ICU stay, IMC stay, and general ward stay. For each patient, all resources used (volumes of use) were assessed on an individual basis and were based on the hospital information system. Volumes of use consisted of, for example, ICU days, IMC days, and general ward days and all diagnostic procedures and medical activities in surgery, laboratory, radiology, and physiotherapy. Surgical interventions on the day of ICU admission were not taken into account. Unit costs were derived from the hospital financial department. Costs per patient resulted from multiplying the unit costs by the frequencies of resources used for the individual patient (unit costs × volumes of use). The cost price of an ICU day (including capacity costs) was €683 and that of a mean general ward day was €90 (range €63 to €146). The cost price of an IMC day was calculated from the costs of medical equipment (capacity costs), nursing staff, and consumable costs (drugs, fluids, nutrition, and disposables) divided by the total annual number of patient days in 2002 (n = 646 days in 8 months). This resulted in a cost price of an IMC day of €505. The costs of the medical staff (non)consultants were not included in the cost calculation. Indirect costs included the general hospital overhead, which was allocated to the direct costs as an overall percentage of 35%, according to the Dutch guidelines for cost calculation [18]. All costs are presented in euros (for 2002) (€1 = US $0.89).

### Statistics

First, we compared the demographic and patient admission characteristics of the pre-IMC and IMC periods by means of the Mann-Whitney test (for continuous variables) and the chi-square test (for categorical data). The total costs of hospital stay were checked for normality of distribution with the Kolmogorov-Smirnov test. If the distribution was not normal, a ^10^log-based transformation was used. Next, an independent group Student *t *test was applied to the cost difference of the pre-IMC and IMC periods. Then, we performed a multiple regression analysis using the periodic change, mortality, type of stay, diagnostic category, and the first TISS-28 score 24 hours after admission (TISS-28 1st day) as predictors of the total costs of hospital stay. Only effects of predictors found by backward elimination which were statistically significant were retained in a 'main effects' regression model. Next, cost effects of first-order interactions between the periodic difference and the other predictors remaining in the 'main effects' model were introduced hierarchically into an extended regression model by forward selection. If one of these interaction effects turned out to be statistically significant, a subgroup regression analysis was performed within the categories of the other relevant factors involved in each interaction to inspect the modification of the periodic difference in cost. The finally found best-fitting model of cost, along with its explained variance, is shown. All data analysis was done with SPSS, versions 12.0 and 15.0 (SPSS Inc., Chicago, IL, USA).

## Results

### Study population

**Figure 1:**
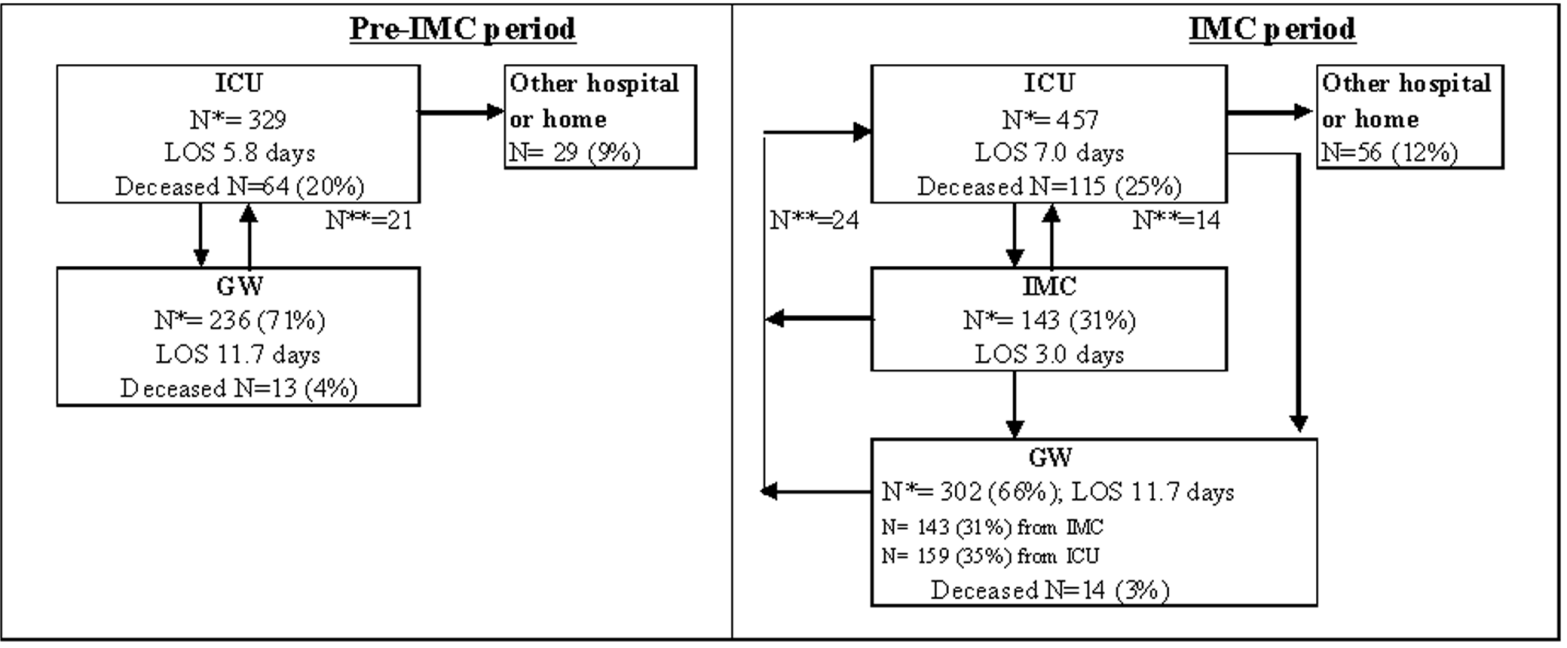
Pre-intermediate care unit (IMC) and IMC period flowchart of intensive care unit (ICU) patients. GW, general ward; LOS, length of stay; N*, number of admissions; N**, number of readmissions.

Figure 1 shows the numbers of admissions to the ICU, the IMC, and the general ward; ICU readmissions; ICU discharges; ICU mean LOS; and the number of deceased patients in the pre-IMC and IMC periods. Table 1 summarises demographic and hospital characteristics for patients in the same two periods.

**Table 1:** Patient and hospital characteristics

	Pre-IMC period	IMC period	*P *value
	n = 329	n = 475	

Number of ICU beds	17	16	
Number of IMC beds	-	6	
Gender, percentage of males	61.2%	59.6%	0.67^a^
Age, mean ± SD, years	57 ± 18.3	56 ± 18.0	0.20^b^
Age, 25%/median/75%, years	43/60/72	44/59/70	
Surgical patients, number (percentage)	175 (53.2%)	277 (60.6%)	0.04^a^
Diagnostic category, number (percentage)			<0.01^a^
Neurosurgical	41 (12.5%)	141 (30.9%)	
Respiratory	88 (26.7%)	87 (19%)	
Gastrointestinal	18 (5.5%)	14 (3.1%)	
Cardiovascular	105 (31.9%)	134 (29.3%)	
Multitrauma	36 (10.9%)	41 (9%)	
Sepsis	16 (4.9%)	20 (4.4%)	
Metabolic/Renal	17 (5.2%)	16 (3.6%)	
Haematological	2 (0.6%)	4 (0.9%)	
Length of stay in the ICU, mean ± SD, days	5.8 ± 9.2	7.0 ± 2.4	0.25^b^
Length of stay in the ICU, 25%/median/75%, days	1/3/6	1/2/8.5	
APACHE II score, mean ± SD (number)	19.5 ± 8.2 (230)	19.9 ± 7.6 (279)	0.44^b^
APACHE II score, 25%/median/75%	13/19/24	14/19/25	
TISS-28 score on 1st day, mean ± SD	28.9 ± 10.8	30.2 ± 11.0	0.16^b^
TISS-28 score on 1st day, 25%/median/75%	21/28/36	22/29/37	
TISS-28 ICU stay, mean ± SD	26.9 ± 9.3	28.6 ± 9.6	0.02^b^
TISS-28 ICU stay, 25%/median/75%	20/26/33	22/28/35	
Mortality in the ICU, number (percentage)	64 (19.5%)	115 (25.2%)	0.06^a^
Mortality after ICU, number (percentage)	13 (4.0%)	14 (3.1%)	0.77^a^

^a^Chi-square tests. ^b^Mann-Whitney tests. APACHE II, Acute Physiology and Chronic Health Evaluation II; ICU, intensive care unit; IMC, intermediate care unit; SD, standard deviation; TISS-28, Therapeutic Intervention Scoring System with a median value of 28.

There were statistically significant differences in the proportion of surgical patients, diagnostic categories, and TISS-28 ICU stay between ICU patients in the pre-IMC and IMC periods. In the pre-IMC group, mean (± standard deviation) LOS in the ICU and TISS-28 score of the randomly selected ICU patients were similar to LOS in the ICU (6.2 ± 17.8 days) and TISS-28 1st day (28.6 ± 10.4) of the total population of the pre-IMC patients (n = 795; *P *= 0.2 for LOS in the ICU; *P = *0.6 for TISS-28 1st day). This indicates that the random sample was representative of the total population of patients.

During the study period, ICU costs showed a tendency to increase after the introduction of the IMC. Total mean hospital cost per stay for ICU patients increased significantly from a mean pre-IMC amount of €12,961 per patient to €16,513 per patient in the IMC period (*P *= 0.01; n = 786). Table 2 shows the costs specified for ICU, IMC, and general ward stays in both the pre-IMC and IMC periods. During the ICU stay, the cost per patient increased by €3,552, which was not significant. Table 2 also shows the hospital costs of some relevant factors studied. There was a significant difference in total hospital costs after opening of the IMC by the group of patients with an LOS in the ICU of more than a week (*P *< 0.001), in non-readmissions to the ICU (*P *= 0.02), and in the group with a TISS score on the first day in the ICU higher than the median score (*P *= 0.01).

**Table 2:** Means, standard deviations, and numbers of patients for hospital cost, also broken down for some relevant factors (n = 786)

Costs	Pre-IMC period		IMC period		*P *value	*P *value interaction
	Number	Mean (SD)	Number	Mean (SD)		

Total hospital stay	329	12.961 (14.530)	457	16.513 (19.324)	0.01	
ICU stay	329	10.017 (13.317)	457	13.020 (17.659)	0.07	
IMC stay	0	-	143	2.612 (4.060)	-	
General ward stay	236	4.105 (5.924)	304	4.065 (5.095)	0.83	
LOS in the ICU of <1 week	155	5.738 (5.028)	231	6.965 (5.922)	0.11	
LOS in the ICU of >1 week	174	19.396 (17.010)	226	26.272 (23.058)	<0.001	0.13
LOS in ward of <1 week	177	9.734 (11.125)	240	13.533 (18.476)	0.01	
LOS in ward of >1 week	152	16.719 (14.045)	217	19.808 (19.743)	0.35	0.22
Readmissions to ICU	21	27.326 (18.558)	38	30.818 (5.627)	0.44	
Non-readmissions to ICU	308	12.130 (13.849)	419	17.499 (847)	0.02	0.99
Surgical patients	175	14.586 (14.162)	277	18.091 (18.483)	0.06	
Non-surgical patients	154	11.115 (14.766)	180	14.085 (20.365)	0.31	0.79
Deceased in hospital	77	14.444 (18.540)	129	18.292 (19.970)	0.10	
Non-deceased in hospital	252	12.509 (13.073)	328	15.813 (19.049)	0.06	0.47
TISS score on 1st day < median	165	9.770 (11.125)	220	10.608 (13.140)	0.62	
TISS score on 1st day > median	149	17.112 (17.138)	230	22.514 (22.450)	0.01	0.14

The *P *value concerns the differences between pre-IMC and IMC periods. The *P *value interaction tests whether the differences of studied relevant factors change from the pre-IMC period to the IMC period. In both tests, ^10^log-transformed costs were analysed. ICU, intensive care unit; IMC, intermediate care unit; LOS, length of stay; SD, standard deviation; TISS, Therapeutic Intervention Scoring System.

A number of variables and factors were introduced into a forced-entry multiple regression model in an effort to explain the cost increase. When introducing predictors as 'patient mortality during ICU stay', 'type of stay' (surgical versus non-surgical), 'TISS-28 1st day', LOS at the ICU, subsequent LOS at the ward, and 'diagnostic category' (as a set of eight dummy variables using metabolic problems as the omitted reference category) into the regression model already containing the 'period' factor (pre-IMC versus IMC), it turned out that all of these predictors made a statistically significant contribution to the prediction of total hospital cost per stay for ICU patients, except 'patient mortality' (Table 3). The higher the TISS-28 score, the higher the costs were (*P *< 0.001), and surgical patients were generally more expensive than non-surgical ones (*P *< 0.001). Patients having a lengthy stay at the ward after the ICU had significantly higher total costs and, next to this, patients having a lengthy stay at the ICU itself also had higher total costs than at the ward (*P *< 0.001).

**Table 3:** Results of final model multiple regression analysis total cost per stay as a 'dependent' outcome variable

Number = 764 Variance explained = 0.680	Unstandardised coefficients		Standardised coefficients		
Predictors	B	Standard error	Beta	*t*	Significance

Period (before → after IMC)	-0.077	0.054	-0.087	-1.420	(0.156)^a^
TISS-28 score on 1st day	0.826	0.152	0.207	5.443	(<0.001)^a^
Surgical (no → yes)	0.183	0.024	0.207	7.714	<0.001
LOS in the ICU	0.025	0.001	0.688	17.307	(<0.001)^a^
LOS in ward	0.016	0.002	0.616	9.940	(<0.001)^a^
Primary organ failure (reference category is metabolic)					
1. Metabolic → cardiovascular	0.066	0.076	0.070	0.871	0.384
2. Metabolic → multitrauma	0.129	0.080	0.088	1.603	0.109
3. Metabolic → gastrointestinal	0.143	0.086	0.064	1.667	0.096
4. Metabolic → haematological	0.324	0.125	0.066	2.589	0.010
5. Metabolic → renal	0.141	0.093	0.050	1.522	0.129
6. Metabolic → neurologic/neurosurgical	0.120	0.076	0.116	1.580	0.115
7. Metabolic → respiratory	0.152	0.075	0.145	2.022	0.044
8. Metabolic → sepsis	0.081	0.085	0.039	0.955	0.340
TISS × period	0.470	0.178	0.184	2.634	0.009
LOS in the ICU × period	-0.005	0.002	-0.135	-3.161	0.002
LOS in ward × TISS-28	-0.022	0.005	-0.289	-4.526	<0.001
Constant	3.220	0.086		37.259	<0.001

^a^*P *values within brackets should not be taken into account as the predictors involved are also present in an interaction effect. Predictors before the arrow have a score of 0, those after the arrow have a score of 1. In the test, ^10^log-transformed costs were analysed. ICU, intensive care unit, IMC, intermediate care unit; LOS, length of stay, TISS-28, Therapeutic Intervention Scoring System with a median value of 28.

Next to this, some diagnostic categories had a statistically significant relationship with total cost. Haematology patients and patients with respiratory problems turned out to have higher costs than patients with metabolic problems. In a 'main effects model' regression analysis, next to the five statistically significant predictors of costs ('TISS-28', 'type of stay', 'length of stay at ICU', 'remaining length of stay at ward', and 'diagnostic category'), the factor pre-IMC and IMC period 'period' was always included. It turned out that the cost in total which was statistically significant in the original *t *test was no longer significant (*P *= 0.173). So the main effects model regression analysis showed no significant difference in total hospital cost between pre-IMC and IMC period controlled for the predictors of costs. Next, effect modification of these predictors can be tested within an extended model using the five first-order interactions involving 'period' in the model. There were only two statistically significant first-order interactions involving 'costs' and 'period': namely, a positive one with the 'TISS-28 score' (*P *= 0.009) and a negative one with the 'length of stay at the ICU' (*P *= 0.002). This means that total costs for patients with relatively high TISS-28 scores were significantly higher in the IMC period compared with the pre-IMC period, but this was not the case for the patients with scores below the median. The other interaction effect showed that, for patients with 3 or more ICU days, costs have significantly risen in the IMC period compared with the pre-IMC period, whereas they remain the same for patients having only 1 or 2 days in the ICU. In testing for the remaining 10 first-order interactions not involving 'period', only one turned out to be statistically significant. It involves the differential effect that the 'remaining length of stay in ward' has on the total costs given the 'admission TISS-28' score. For both periods, a general effect strengthening of the 'TISS-28 score' on total costs is taking place for patients with a ward stay up to 1 week compared with the patients with a ward stay of more than 1 week. The definite, extended regression model results are reported in Table 3.

## Discussion

The ICUs generally consume a considerable proportion of hospital resources [19,20]. Evaluation of cost and cost-effectiveness is vital for assessing the impact of implementing new strategies for improved intensive care [21,22]. This study shows that, despite a lower cost price of an IMC day, total hospital costs were not reduced after the introduction of the IMC. By introducing the IMC, the total absolute hospital cost per patient increased significantly from €12,961 (± €14,530) in the pre-IMC period to €16,513 (± €17,718) in the IMC period. This could be explained by a significantly higher LOS in the ICU and TISS-28 1st day and not by the introduction of the IMC itself. The patients in the IMC period were more severely ill because of greater requirements for care (as judged by the TISS-28 score) and a longer length of ICU stay. Still, specific groups of patients had higher costs in the IMC period compared with the pre-IMC period. Patients with more than 3 days of ICU stay had a higher cost in the IMC period compared with the pre-IMC period.

A limited number of studies have addressed the impact of the introducing an IMC. One study shows reduced costs without a negative impact on outcome [7]. Another randomised study finds no overall difference, although costs to produce a survivor were reduced in the IMC [8]. Both studies, however, estimated solely specialised care costs. This is the first study to assess the effect on total hospital costs in a prospective cohort study.

Several factors may explain the absence of reduced total hospital costs despite reduced costs for intermediate care patients. Although the IMC patients accounted for 24% of the patients admitted for specialised care, they consume a relatively small proportion of the ICU costs. The overall impact of cost reduction by treating the low-risk patients in the IMC instead of the ICU thus may not be relevant. In the study of Zimmerman and colleagues [4], low-risk ICU patients consumed only 8.5% of the total ICU costs, which is similar to our results.

The calculated costs per individual TISS unit increased from €347 to €431 during the study period. This may be due to increases in radiographs, laboratory tests, and costs of prescribed medications.

The relatively high cost price of an IMC day is related to the low occupancy rate in the IMC in its first year and a high nurse-to-patient ratio in the IMC. We expect the average cost to decline with increasing occupancy rates, resulting in lower total hospital costs per patient. For example, the total IMC cost was €326,230 in the 8-month IMC period. The IMC has six beds and the potential for 1,470 patient days/8 months. An IMC occupancy rate of 85% resulted in a cost price of an IMC day of €261. The reason for the low occupancy rate in the IMC was the high workload of the medical and nursing teams. The same number of critical care physicians had to cover this expansion of critical care services.

## Conclusion

The cost-effectiveness of an IMC is relative to the size of the unit and hence to the number of beds that are staffed and the number of patients who can be treated [23]. By far the most expensive item on the budget is nursing costs. Overall costs per patient can be reduced considerably if the size and utilisation of an IMC are optimal. In conclusion, after the opening of the IMC, more surgical patients, greater requirements of therapeutic interventions on the ICU admission day, and longer ICU stay in patients explained the increase in hospital costs, rather than the introduction of the IMC itself.

## Key messages

• Length of intensive care unit stay was the most important factor in the increase in total hospital cost.

• Difference in case mix, reflected in more surgical patients and a higher TISS-28 (Therapeutic Intervention Scoring System) score on admission day, explained the increase in total hospital cost after introducing an intermediate care unit (IMC).

• Optimal size and utilisation of the IMC may reduce the total hospital cost per patient.

## Abbreviations

APACHE II = Acute Physiology and Chronic Health Evaluation II; ICU = intensive care unit; IMC = intermediate care unit; LOS = length of stay; TISS-28 = Therapeutic Intervention Scoring System; TISS-28 1st day = first Therapeutic Intervention Scoring System score 24 hours after admission.

## Competing interests

The authors declare that they have no competing interests.

## Authors' contributions

BCJS participated in study conception, study design, data acquisition, data analysis and interpretation, and drafting of manuscript. CDD and FHMN participated in statistical and cost analyses and in editing the manuscript. GvM and GR participated in editing the manuscript. MP participated in methodology and in editing the manuscript. All authors read and approved the final manuscript.
